# Mercury-induced toxicity of rat cortical neurons is mediated through N-methyl-D-Aspartate receptors

**DOI:** 10.1186/1756-6606-5-30

**Published:** 2012-09-14

**Authors:** Fenglian Xu, Svetlana Farkas, Simone Kortbeek, Fang-Xiong Zhang, Lina Chen, Gerald W Zamponi, Naweed I Syed

**Affiliations:** 1 Department of Cell Biology & Anatomy, University of Calgary, Alberta, Canada; 2 Physiology & Pharmacology, Hotchkiss Brain Institute, Faculty of Medicine, University of Calgary, Alberta, Canada

**Keywords:** Mercury Chloride, Rat cortical neurons, Toxicity, MK 801, NMDA receptor, Excitotoxicity, Cytoskeleton

## Abstract

**Background:**

Mercury is a well-known neurotoxin implicated in a wide range of neurological or psychiatric disorders including autism spectrum disorders, Alzheimer’s disease, Parkinson’s disease, epilepsy, depression, mood disorders and tremor. Mercury-induced neuronal degeneration is thought to invoke glutamate-mediated excitotoxicity, however, the underlying mechanisms remain poorly understood. Here, we examine the effects of various mercury concentrations (including pathological levels present in human plasma or cerebrospinal fluid) on cultured, rat cortical neurons.

**Results:**

We found that inorganic mercuric chloride (HgCl_2_ –at 0.025 to 25 μM) not only caused neuronal degeneration but also perturbed neuronal excitability. Whole-cell patch-clamp recordings of pyramidal neurons revealed that HgCl_2_ not only enhanced the amplitude and frequency of synaptic, inward currents, but also increased spontaneous synaptic potentials followed by sustained membrane depolarization. HgCl_2_ also triggered sustained, 2–5 fold rises in intracellular calcium concentration ([Ca^2+^]_i_). The observed increases in neuronal activity and [Ca^2+^]_i_ were substantially reduced by the application of MK 801, a non-competitive antagonist of N-Methyl-D-Aspartate (NMDA) receptors. Importantly, our study further shows that a pre incubation or co-application of MK 801 prevents HgCl_2_-induced reduction of cell viability and a disruption of β-tubulin.

**Conclusions:**

Collectively, our data show that HgCl_2_-induced toxic effects on central neurons are triggered by an over-activation of NMDA receptors, leading to cytoskeleton instability.

## Background

Mercury is a potent environmental contaminant that exerts toxic effect on a variety of vital organs in the human body. It exists in three predominant forms: elemental (Hg^0^), organic (such as methylmercury -MeHg), and inorganic (mercuric chloride - HgCl_2_) mercury [1]. The major sources of mercury load in humans are food contamination, drug and vaccine preservatives, dental amalgams, or occupational exposure [2]. Both acute and chronic exposure to mercury is also known to cause a variety of neurological or psychiatric disorders [3-6]. The central nervous system (CNS) is one of the most vulnerable organs affected by mercury toxicity. Within the CNS, two of the most often affected areas are the cerebral cortex [7,8] and the cerebellum [9,10]. While the mechanisms underlying mercury-induced toxicity remain largely unknown, several studies have shown toxic effects of organic MeHg on the CNS as it can easily cross the blood–brain barrier and accumulate in the brain at high concentrations [1,11]. MeHg has been reported to interact with a wide range of cellular targets and affect multiple cellular functions. These toxic effects include, but are not limited to, the inhibition of neuronal ion channels [12-14], disruption of presynaptic transmitter release and postsynaptic receptor function [15-17], damage to neuronal cytoskeleton components and DNA structures [8,18,19], and alteration of Na^+^/K^+^ ATPase and mitochondrial function [20,21] (also see review [22]). Moreover, a large body of recent evidence has also drawn the link between mercury and glutamate-mediated excitotoxicity. For instance, mercury has been shown to affect several aspects of glutamatergic signaling, including the inhibition of glutamate reuptake in astrocyte [23], inhibition of glutamine synthetase activity [24], and an enhancement of spontaneous glutamate release from neurons [25]. These effects result in an increase in glutamate concentration at the synaptic cleft. MeHg has also been found to impact postsynaptic N-Methyl-D-Aspartate (NMDA) receptors. For instance, Basu et al. (2007) have demonstrated that mercury decreases the number of NMDA receptors in several brain regions including the basal ganglia, cerebellum, brainstem, and occipital cortex of mink [26]. In contrast, Ndountse and Chan (2008) have reported that MeHg increased NMDA receptor expression, which in turn may be responsible for neurotoxicity [27]. Another study by Miyamoto et al. (2001) suggested that the enhanced sensitivity of NMDA receptors might contribute to the vulnerability of developing cortical neurons to MeHg neurotoxicity [28]. Although the mercury-induced toxic effects on the nervous tissue have been well documented, the findings are often conflicting and the underlying mechanisms remain poorly defined.

In contrast to MeHg, much less is known about the toxic effect of inorganic HgCl_2,_ despite the fact that it can easily cross the blood–brain barrier and accumulate in the brain at much higher concentrations [29,30]. Moreover, HgCl_2_ is a more reactive form of mercury as it interacts with many macromolecules and exhibits a long latency of neurotoxicity [24,31]. For instance, HgCl_2_ is known to be five times as potent as MeHg in blocking L-type Ca^2+^ channels in hippocampal neurons [32]. It is also a more potent inhibitor of glutamate reuptake and glutamine synthetase activity than MeHg [24]. However, little is known about its actions on the NMDA receptors. In the present study, we first assessed the impact of inorganic HgCl_2_ on neuronal viability and network integrity in both developing and matured rat cortical neurons in cell culture. Next, we investigated the cellular mechanisms underlying HgCl_2_-induced toxic effect by studying the involvement of glutamatergic NMDA receptor activity. Our results show that HgCl_2_ disturbs neuronal network structure and induces cell apoptosis in cortical neurons. These actions involve a degradation of β-tubulin, an important component of the neuronal cytoskeleton, and these effects were evoked by NMDA-receptor function. Inhibition of NMDA-receptor activity by MK 801 not only inhibited HgCl_2_-induced increase in synaptic currents and intracellular Ca^2+^, but also prevented HgCl_2_-induced disassembling of cytoskeleton protein and disruption of neuronal network integrity.

## Results

### HgCl_2_ inhibits neuronal outgrowth and induces degeneration of both developing and mature cortical neurons in culture

**Figure 1:**
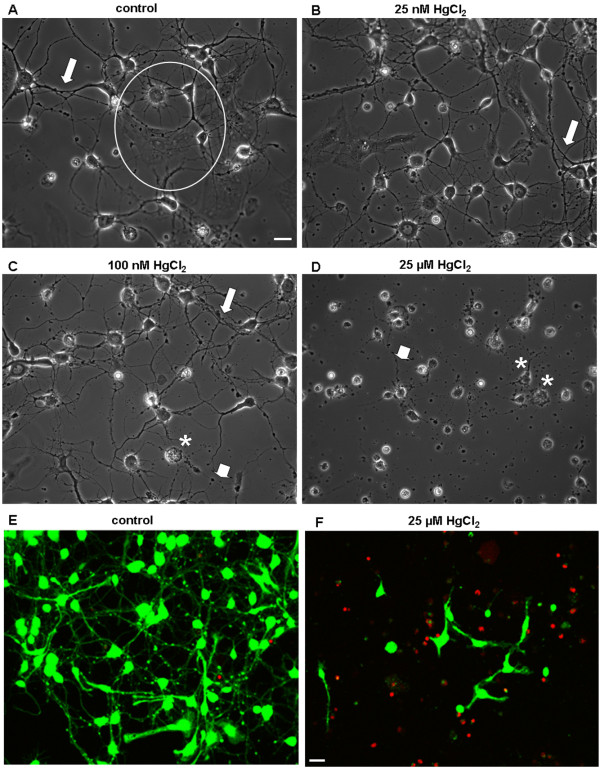
**HgCl**_**2**_** exposure perturbs neurite outgrowth and compromises neuronal viability in cultured cortical neurons.** Rat cortical neurons were grown either in the absence (**A**, control) or presence of HgCl_2_ at 25 nM (**B**), 100 nM (**C**), and 25 μM (**D**) for 3 days. Phase contrast images were taken on day 3. **A**. Cortical culture under control conditions exhibited healthy population of neurons and glial cells (indicated by a circle), exhibiting extensive neurite outgrowth and network formulation (indicated by an arrow). **B**. Cells cultured in the presence of 25 nM HgCl_2_ also developed extensive neurite processes and formed extensive network. **C**. Neuronal populations cultured in the presence of 100 nM of HgCl_2_ underwent apoptotic cell death (indicated by an asterisk) and exhibited collapse or retraction of neurites (indicated by an arrow head). **D**. HgCl_2_ at 25 μM induced cell death (indicated by asterisks) and inhibited neurite extension and network formation (indicated by an arrow). **E** &**F** shows representative confocal images of live/dead assay of cortical neurons cultured either in the absence (**E**) or presence (**F**) of 25 μM of HgCl_2_ for 3 days. Live cell bodies and their neurites were stained with calcein-AM (green), whereas dead cells with compromised membranes were stained with ethidium homodimer-1 (red). Scale bars, 20 μm.

**Figure 2:**
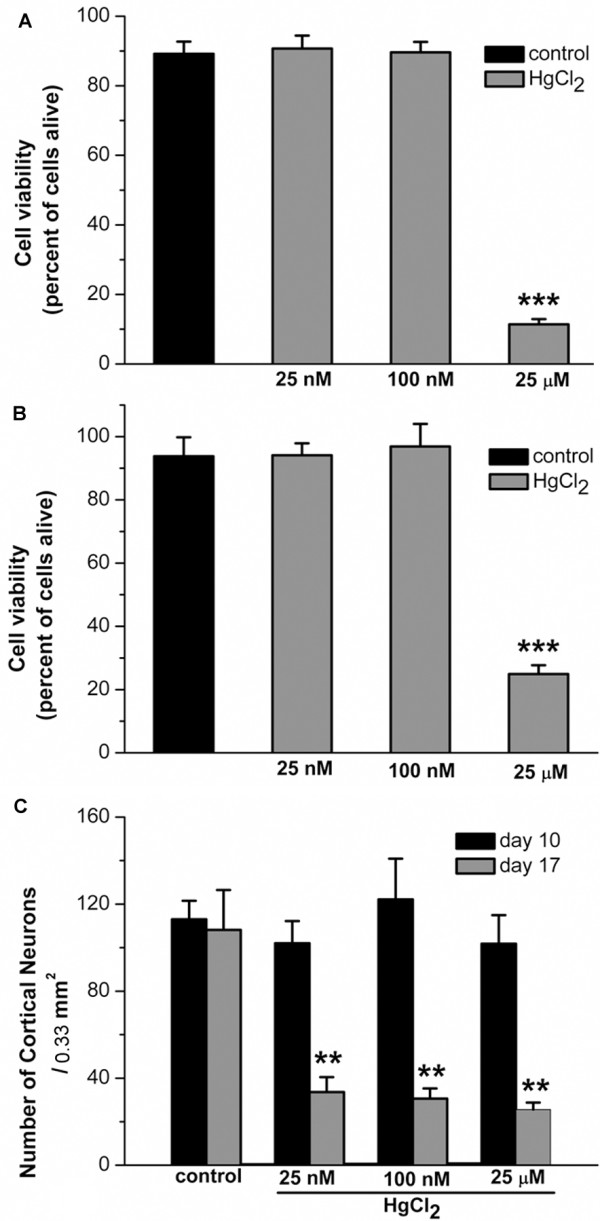
**Statistical data showing the acute and chronic effects of HgCl**_**2**_** on cell viability of developing and mature cortical network. A**. To evaluate the effect of HgCl_2_ on neurite initiation and network formation during the early stage of neuronal development, rat cortical cells were cultured in the absence (control) or presence of HgCl_2_, at 25 nM, 100 nM, and 25 μM, for 3 days. Cell viability/cytotoxicology assay were performed on day 3. The number of live and dead cells in randomly chosen areas of 1 mm^2^ was counted. The percentage of live cells is presented in the bar graph. These data show that cultures in the presence of HgCl_2_ at 25 μM, but not 25 or 100 nM, exhibited significant reduction of cell viability. **B**. To evaluate the acute effect (< 24 hrs) of HgCl_2_ exposure on the newly developed network, 4 day old cortical cultures were exposed to HgCl_2_, at 25 nM, 100 nM, and 25 μM, for 16 to 24 hrs. Subsequent live/dead cell assay showed that acute exposure to higher concentration of HgCl_2_ at 25 μM significantly reduced the cell viability. **C**. To evaluate the chronic effect (7 days) of HgCl_2_ exposure on the neuronal network, cells were grown for 10 days and were maintained for another 7 days in the absence or presence of HgCl_2_. Phase contrast images of neurons in the same area of 0.33 mm^2^ were taken on day 10 and day 17. The statistic data show that the numbers of neurons were significantly reduced by exposure to HgCl_2_ for 7 days at all concentrations examined. Statistical significance was determined using ANOVA (**A** &**B**) and Student’s paired *t*-test (**C**). Post hoc analysis was conducted using Tukey’s test. ** *P* < 0.01. *** *P* < 0.001. Error bars indicate SEM for all figures.

To examine how mercury may impact neuronal outgrowth and network formation between central neurons, we first investigated the effects of HgCl_2_ on neurite ini-tiation and network formation during early outgrowth. To this end, rat neonatal cortical neurons were cultured either in the absence (control) or presence of HgCl_2_ at the concentrations of 25 nM, 100 nM, and 25 μM for 3 days. Neuronal viability, neurite outgrowth and network formation were evaluated 3 days later. The choices of these experimental doses were based on the studies showing that in the Minamata Bay exposure in Japan, the mercury concentration in the brains of patients was found to be around 1.7 μM to 26 μM, whereas in non-exposed controls its levels averaged around 30 nM [33,34]. Additionally, several studies have revealed that mercury levels in brain tissues are 2–10 fold higher in patients with dental amalgam fillings, Alzheimer’s disease, autism spectrum disorders, epilepsy or hydrocephalus [35-38]. Figure 1A shows a typical culture dish grown under control conditions (no mercury) yielding a healthy population of both neurons and glial cells (indicated by a circle) with extensive neurite outgrowth and network formation (indicated by an arrow). Although cells developed extensive neurites and neuronal network in the presence of both 25 nM and 100 nM HgCl_2_ (Figure 1B & C, indicated by arrows), it is interesting to note that HgCl_2_, at 100 nM, appeared to cause cell body damage, abnormal neurite outgrowth, or degeneration of some neurites (Figure 1C, indicated by an asterisk and an arrow head). Cells cultured in the presence of 25 μM of HgCl_2_ however failed to develop neurite processes and hence were devoid of network connectivity (Figure 1D, indicated by an arrow head and asterisks). To quantify the effect of HgCl_2_ on cdead cell assay /dead cell assay (LIVEDEAD® ViabilityCytotoxicity Kit, Invitrogen, see Materials and Methods). Specifically, cells were maintained in culture for 3 days either in the absence or presence of HgCl_2_ at concentrations as described above. On day 3, neurons were incubated and stained with green-fluorescent calcein-AM for 30 mins to indicate intracellular esterase activity (live cells), and red-fluorescent ethidium homodimer-1 to determine the damage to plasma membrane integrity (dead cells) (Figure 1E & F). Fluorescent labeling of live and dead cells was visualized using a confocal microscope (LSM-510) and images were acquired under the same confocal parameter settings using a 10x or 20x objective. The number of live (green) and dead (red) cells in the above culture conditions was counted using imageJ software. Specifically, neurons were counted based on a randomized selection of three areas of 1 mm^2^ under each culture condition. The mean value of cell viability reflected by the percentage of live cells was calculated and compared. Figure 2A showed that in four experiments cell viability under control conditions was 89.2 ± 3.5% (n = 4). There was however no significant difference between 25 nM (90.7 ± 3.7%, n = 4, *P* = 0.352) and 100 nM (89.6 ± 3.0%, n = 4, *P* = 0.914) of HgCl_2_. HgCl_2_ used at the concentration of 25 μM did, however, significantly (11.4 ± 1.5%, n = 4, *P* < 0.0001) compromised cell viability compared to control (Figure 2A).

We next sought to determine whether the established cortical neuronal network at early stages of cell culture could be affected by shorter exposure (< 24 hrs) to HgCl_2_ at different concentrations. To do this, cells were maintained in culture for 4 days and allowed to exhibit neurite outgrowth and network connectivity. Neurons were then cultured in medium containing either no HgCl_2_ (as control) or HgCl_2_ at 25 nM, 100 nM or 25 μM for 16–24 hrs. To evaluate the effects of mercury on neuronal network and cell viability, neurons were again stained with calcein-AM (live) and ethidium homodimer-1 (dead) after treatment. The protocol for live/dead cell counting and cell viability calculation was as described in Figure 2A. As shown in Figure 2B, HgCl_2_ at lower doses such as 25 nM (93.8 ± 6%, n = 4, *P* = 0.072) and 100 nM (94.1 ± 3.8%, n = 4, *P* = 0.254) did not affect the established network and cell survival as compared with control (96.9 ± 7.1%, n = 4). However, a shorter (16–24 hrs) exposure of cells to 25 μM of HgCl_2_ not only remarkably disrupted the neuronal physical connectivity, but also significantly reduced the cell viability (24.9 ± 2.8%, n = 4, *P* < 0.001) (Figure 2B).

Finally, we sought to determine whether longer-term exposure (>7 days) of cells to HgCl_2_ could affect fully developed neurons and their network. To test this, cortical cells were first allowed to grow in culture for 10 days and establish outgrowth and extensive networks. Cells were subsequently treated with HgCl_2_ at 25 nM, 100 nM and 25 μM and maintained in culture for another 7 days. Neuronal viability and network formation were examined at day 17. We found that at this stage, the cortical neurons exhibited extremely dynamic neuronal outgrowth pattern, and the active growth of glial cells formed enormous layers in the culture dish. The extensive background labeling of glial cell layers made the survival assay, particularly the counting of live cells, very difficult. We therefore opted to evaluate the effect of HgCl_2_ on neuronal viability using phase contrast images. Specifically, cortical neurons were cultured on glass cover slips with a grid (Bellco Glass, aid for counting). Phase contrast images were taken before exposure to HgCl_2_ on day 10 and after an addition 7-day exposure to HgCl_2_ (i.e., on day 17). The average numbers of neurons were calculated based on a randomized selection of five different areas of 0.33 mm^2^ in each dish. With the aid of the grid on cover slips, we were able to take images of the same five areas in each dish on day 10 and day 17. The mean numbers of survived neurons were compared between day 10 and day 17. Neurons were identified based on their size, shape, arrangement and number of processes. Our results showed that 7-day exposure to HgCl_2_, at all concentrations examined, caused a significant reduction in the rate of neuronal survival. Specifically, control cultures had on average 113 ± 8.5 neurons/0.33 mm^2^ on day 10 and it did not change after 7 days in culture (108.2 ± 18.3, n = 5, *P* = 0.842). Cultures exposed to 25 nM HgCl_2_ for 7 days had a substantial reduction in the number of neurons (33.6 ± 6.9, n = 5, *P* = 0.005) as compared with that before HgCl_2_ exposure on day 10 (102 ± 10.2, n = 5). Similarly, HgCl_2_ at 100 nM (from 122.2 ± 18.7 to 30.6 ± 4.7, n = 5, *P* = 0.003) and 25 μM (from 101.8 ± 13.1 to 25.6 ± 3.2, n = 5, *P* = 0.002) caused significant decrease in cell viability (Figure 2C). These data indicated that chronic exposure to HgCl_2_ at concentrations, as low as 25 nM to 100 nM, caused detrimental effects on central neurons even at a more matured developing stage. It is, however, important to note that because the mercury-induced effects are not neuron-type specific, the significant reduction in the number of neurons at all concentrations examined might also involve a secondary toxic effect involving the glial cells. Specifically, mercury toxicity to glial cells may prevent astrocytes from forming a firm “bottom layer” which plays an essential role for neuronal outgrowth and survival under our culture condition. The toxic impact of mercury on glial cells may have thus exasperated neuronal loss in our culture condition. However, our experiments together with previous studies warn against the potential effects of chronic low-dose mercury exposure [39,40].

Based on the fact that HgCl_2_ used at 25 μM consistently caused neuronal damage to both developing and more mature neurons during short-term and long-term exposure, we next investigated the mechanisms underlying the toxic effect of HgCl_2_ on cortical neurons using the concentration of 25 μM in all subsequent experiments, unless stated otherwise.

### HgCl_2_-induced cell damage is associated with loss of cytoskeleton structures

**Figure 3:**
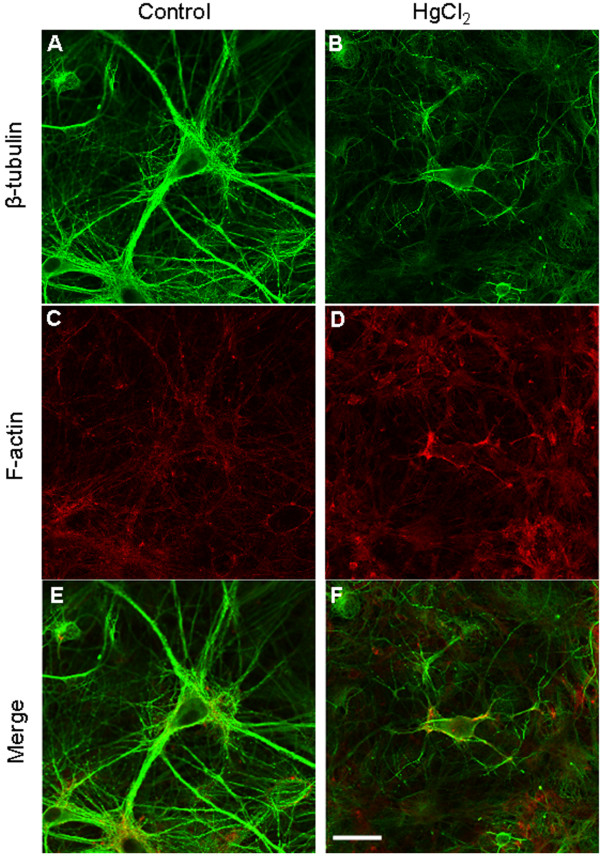
**Exposure of HgCl**_**2**_** compromised cytoskeleton components, mainly the β-tubulin, in cortical neurons.** Cells were first cultured for 4 to 10 days and allowed to develop neurite and networks and were then exposed to 25 μM of HgCl_2_ for up to 24 hrs. Cultures were subsequently fixed and stained with antibodies against β-tubulin and rhodamine-phalloidin (**A**-**D**). **E** &**F** are the merged confocal images of β-tubulin and F-actin staining. The fluorescence intensity of β-tubulin, and not F-actin was reduced in neurons that were treated with 25 μM of HgCl_2._ Scale bar, 25 μm.

The architecture and survival of the central neurons rely significantly upon their cytoskeleton components, specifically the actin filament (F-actin) and microtubules such as β-tubulin. Any disturbance of these dynamic structures or their polymerization/depolymerization status due to environmental or genetic factors could compromise cell survival [41-43]. Based on our observations that HgCl_2_ induces cell damage and retraction of neuronal processes, we hypothesized that HgCl_2_ toxicity may involve disruption of neuronal cytoskeleton proteins such as the F-actin and β-tubulin. To test this hypothesis, cortical neurons were first cultured for four to ten days, which allowed for the development of neurite outgrowth and network connectivity. Cells were then exposed to culture medium containing either no HgCl_2_ (as control) or HgCl_2_ at 25 μM for 24 hrs. The preparations were then fixed with 4% paraformaldehyde and stained with anti β-tubulin antibody and rhodamine-phalloidin (to label F-actin). As shown in Figure 3, the pyramidal cortical neurons in control condition (A) exhibited bigger cell bodies with multiple healthy processes and extensive network. In contrast, neurons treated with HgCl_2_ (B) appeared to be much smaller in size with degraded neurite and compromised network. Accordingly, a strong fluorescent labeling of cell bodies and neurites with both β-tubulin (A) and F-actin (C) antibodies were observed in control neurons. The cells exposed to HgCl_2_ at 25 μM, however, remarkably lost the fluorescent intensity of β-tubulin (B) although there was no apparent change in the intensity of F-actin staining (D). These results clearly demonstrated that the β –tubulin protein is most likely affected by HgCl_2_-induced toxicity in the CNS. The reduction of overall intensity of β –tubulin is likely due to HgCl_2_-induced degeneration of neurons rather than cell death as the level of F-actin intensity remained unaffected by HgCl_2._ One would need to be cautious, however, that these experiments may not preclude the possibility that mercury exposure might also change the conformation of β –tubulin altering access of the primary antibody to the epitope. Future investigation and analysis using western blot techniques are still required for further validation of these data.

### HgCl_2_ triggers sustained intracellular Ca^2+^ rise in cortical neurons, which is blocked by NMDA receptor antagonists

**Figure 4:**
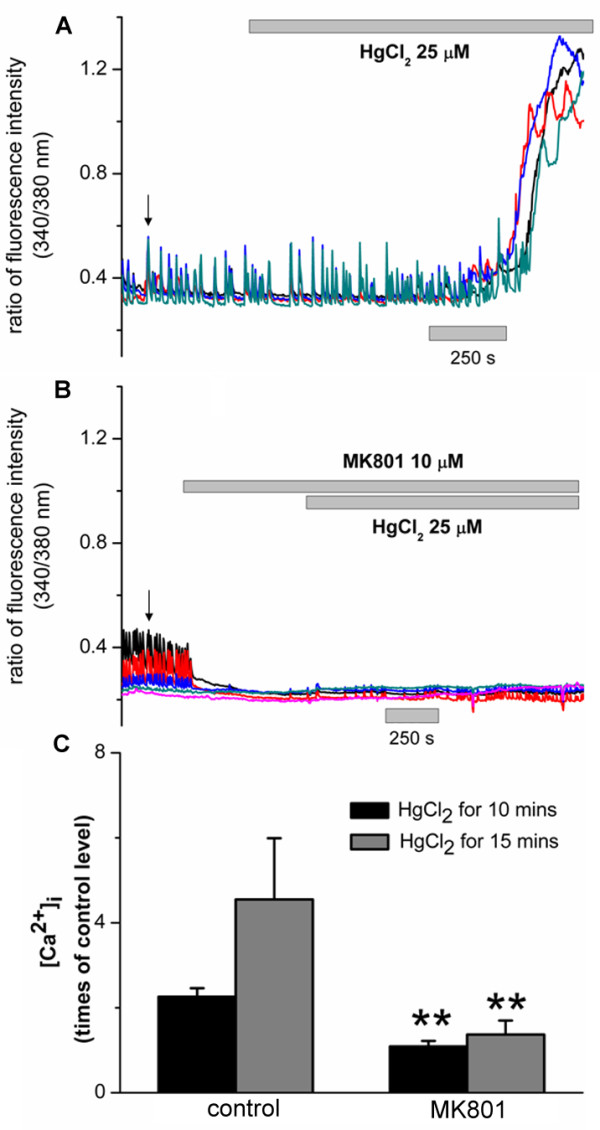
**HgCl**_**2**_** induced sustained elevation of intracellular Ca**^**2+**^** concentration ([Ca**^**2+**^**]**_**i**_**) in cortical neurons and this rise in [Ca**^**2+**^**]**_**i**_** was inhibited by the NMDA receptor antagonist MK 801. A**. Representative traces of changes in fura-2 fluorescence ratio before and after the application of HgCl_2_ (25 μM) in four different cortical neurons (shown by red, green, blue and black traces). Cells were loaded with fura-2 AM and the ratio of fluorescence intensity, at the 340 and 380 nm excitation wavelengths, was measured **B**. Representative traces of fura-2 fluorescence ratio recorded before and after application of HgCl_2_ (25 μM) in four different cortical neurons (shown by red, green, blue and black traces) that were previously exposed to MK 801 (10 μM), an antagonist of NMDA receptors. **C**. Fold- rises in fura-2 fluorescence ratio induced by HgCl_2,_ after 10 and 15 mins of exposure time, in the absence or presence of MK 801. Note that, HgCl_2_-induced rise in [Ca^2+^]_i_ in MK 801-treated neurons was significantly smaller than that in control cells in the period of 10 mins and 15 mins. Arrows indicate the presence of spontaneous Ca^2+^ transients in cortical neurons under resting conditions.

Recent studies have linked the degeneration of cytoskeleton structures to glutamatergic excitoxicity. Specifically, over stimulation of NMDA receptors and the subsequent overloading with Ca^2+^ have been shown to cause rapid disruption of established cytoskeleton components or inhibition of the assembly of new cytoskeleton elements such as microtubules, F-actin and neurofilament proteins in a variety of preparations [44-46]. Moreover, NMDA receptors have been shown to play a pivotal role in mercury-induced neurotoxicity [19,28]. We next sought to determine whether HgCl_2_ affects cortical Ca^2+^ homeostasis through NMDA receptors. To this end, Ca^2+^ ratiometric experiments using Fura-2 AM were performed on cortical neurons. Neurons were maintained in culture for about five to ten days and subsequently loaded with 5 μM of Fura-2 AM for 30 mins at 37°C. Cells were then washed with normal saline containing free Mg^2+^ and 3 μM of glycine in order to activate NMDA receptors at the resting level. Ca^2+^ images and the ratio of Fura-2 fluorescence intensity at 340 nm and 380 nm (reflection of intracellular Ca^2+^ levels) were acquired prior to and post exposure to HgCl_2_ at 25 μM. Representative traces of four different cells are shown in Figure 4A (red, green, black and blue traces). As shown in Figure 4A, intracellular Ca^2+^ transients were recorded before HgCl_2_ (indicated by an arrow). After local application of HgCl_2_, the amplitude and frequency of calcium transients were augmented. After 5 to 10 mins of HgCl_2_ exposure, sustained rises in intracellular Ca^2+^ were observed in almost all cells examined (Figure 4A). The ratio of Fura-2 fluorescence intensity increased by 2.26 ± 0.2 times (n = 8) after 10 mins of HgCl_2_ application and 4.55 ± 1.44 (n = 11) times compared to control levels after 15 mins of HgCl_2_ application (Figure 4C, two columns on the left panel). Next, we examined the involvement of NMDA receptor activity in HgCl_2_-induced rise in intracellular Ca^2+^. To do this, the basal Ca^2+^ levels were first monitored for 5 mins and again intracellular Ca^2+^ transients were recorded in control conditions (Figure 4B, indicated by an arrow). The cells were then exposed to 10 μM of the NMDA receptor antagonist MK 801 for 5 to 10 mins. Note that upon application of MK 801, the intracellular Ca^2+^ transients were almost completely abolished indicating an involvement of tonic NMDA receptor activity in normal Ca^2+^ oscillations. It is also important to note that subsequent application of HgCl_2_ (25 μM) in the continued presence of MK 801, failed to induce rises in Ca^2+^. In MK 801 treated neurons, the fluorescence ratio in the continued presence of MK 801 was 1.09 ± 0.13 (n = 11) times after 10 mins of HgCl_2_ exposure and 1.37 ± 0.33 times (n = 8) after 15 mins of HgCl_2_ exposure (Figure 4C, two columns on the right panel). The rise in intracellular Ca^2+^ in the presence of MK 801 was significantly smaller than that of MK 801 (Figure 4C). These results indicate that NMDA receptor activity may play a crucial role in HgCl_2_-induced alteration of intracellular Ca^2+^ homeostasis, which might contribute to HgCl_2_-induced disturbance of cytoskeleton network.

It is important to note that HgCl_2_-induced sustained elevations of [Ca^2+^]_i_ occurred simultaneously in groups of cells that were measured as shown in Figure 4A. This led us to propose that HgCl_2_ may initially affect the synaptic activity of cortical neurons by acting through their NMDA receptors. These changes in neuronal excitability may in turn alter the status of dynamic or balanced excitability within a network or their neighboring cells.

### HgCl_2_ increased synaptic inward currents and cause sustained membrane depolarization in pyramidal cortical neurons through NMDA receptors

**Figure 5:**
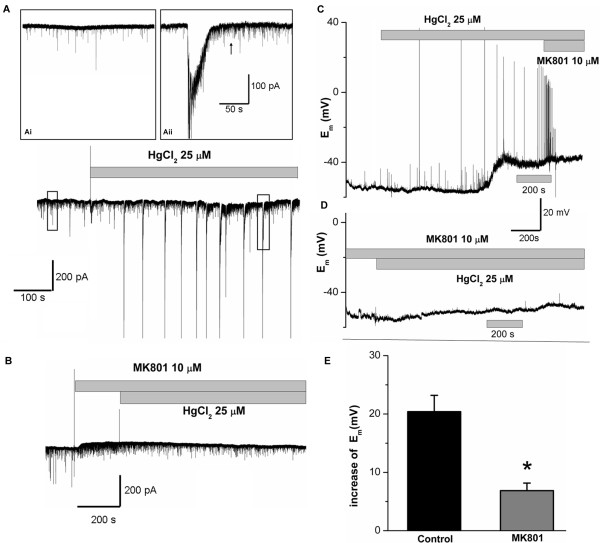
**HgCl**_**2**_** enhanced spontaneous inward currents and caused sustained membrane depolarization in pyramidal cortical cells. A**. A representative trace of spontaneous synaptic currents recorded before and after the application of HgCl_2_ (25 μM) from a pyramidal neuron. The cell was held at the potential of −70 mV. Spontaneous inward currents were recorded before (Ai) and during the application of HgCl_2_. Both the amplitude and frequency of inward current were substantially increased. Robust small, spontaneous events followed the big amplitude of currents as shown in the insert (Aii, indicated by an arrow). **B**. A representative trace of synaptic currents recorded before and after HgCl_2_ exposure in the presence of 10 μM of MK 801. Note that application of MK 801 prevented HgCl_2_ from inducing increase of synaptic currents indicating that NMDA currents was involved in HgCl_2_ action on cortical neurons (n = 7). **C**. Membrane potential of a pyramidal cell recorded under current-clamp mode before and after the application of HgCl_2_ at 25 μM. HgCl_2_ increased the spontaneous electrical activity and triggered sustained rise in membrane potentials. Subsequent application of MK 801 (10 μM) abolished HgCl_2_-induced firing of action potentials but did not affect the sustained membrane depolarization. **D**. A representative trace of membrane potential recorded from a pyramidal cell pretreated with MK 801 for 10 mins before and after HgCl_2_ exposure,. Pretreatment of cells with MK 801 greatly reduced HgCl_2_ - induced increase in action potential firing and sustained membrane depolarization. **E**. Statistical data shows that the increased amplitude of sustained membrane depolarization by HgCl_2_ (25 μM) in MK 801-treated cells is significantly smaller than that in control cells.

NMDA receptor-mediated synaptic activity plays an important role in early neuronal development and also in the later stages of synaptic maturation [47-50]. To further determine the role of NMDA receptors in HgCl_2_-induced toxicity, we next explored whether HgCl_2_ acts to alter NMDA receptor-mediated synaptic activity such as synaptic currents or membrane discharges. We first tested whether HgCl_2_ affected spontaneous synaptic currents in pyramidal neurons. To test this, whole cell voltage-clamp experiments were performed on pyramidal neurons in culture for 7 to 15 days and spontaneous inward currents were recorded by holding the cells at −70 mV. As shown in Figure 5A, spontaneous inward currents were recorded in pyramidal cortical neurons under control condition as shown in the insert (Ai). Upon local application of HgCl_2_ (25 μM), large amplitude inward currents were elicited. It is important to note that HgCl_2_-elicited inward currents were often followed by high frequency, spontaneous postsynaptic events, which lasted for a long period (see insert Aii, indicated by an arrow). To test the involvement of NMDA receptors in HgCl_2_-induced increase in synaptic currents, the inward currents were recorded from cells that were first exposed to MK 801 to block the NMDA-receptors, and then to both MK 801 and HgCl_2_. Figure 4B shows that spontaneous synaptic currents were again recorded under control conditions. Application of MK 801 eliminated events with bigger amplitudes indicating the involvement of NMDA-currents in these events. The remaining synaptic events in the presence of MK 801 are likely the non-NMDA currents. Interestingly, in the continued presence of MK 801, HgCl_2_ failed to further enhance synaptic currents that were observed in Figure 4A, while the frequency and amplitude of non-NMDA currents remained unchanged by HgCl_2_. These experiments suggest that HgCl_2_ mainly increased the NMDA-currents in pyramidal cortical neurons.

The above data suggest that HgCl_2_-induced increase in synaptic inputs in pyramidal cortical neurons may in turn change the membrane excitability in these neurons. To test this possibility, we next monitored the membrane potential changes by performing whole-cell current-clamp experiments on pyramidal cortical neurons. Figure 5C shows a representative membrane potential trace recorded from a pyramidal neuron before and after the application of 25 μM of HgCl_2_. In this particular cell, the resting membrane potential of the cell was about −56 mV. Application of HgCl_2_ initially enhanced the spontaneous synaptic discharge and triggered firing of action potentials followed by sustained membrane depolarization to a potential of around −42 mV. Interestingly, subsequent application of MK 801 (10 μM) did abolish the firing of action potentials, but did not affect the sustained membrane depolarization indicating the involvement of secondary-mechanism in the sustained depolarizing components. It is interesting to note that pre-incubation of cells with MK 801 at 10 μM for about 10 mins almost completely prevented HgCl_2_ from triggering action potentials and eliciting large sustained membrane depolarization as shown in Figure 5D (n = 7). Specifically, we found that HgCl_2_ failed to induce an increase in synaptic potentials and firing of action potentials after 10 to 15 mins of exposure time, although a small elevation of membrane potential was still detectable in some cells. The mean amplitudes of sustained membrane depolarization either in the absence (control) or presence of MK 801 is shown in Figure 5E. These data reveal that the mean increase in membrane potentials of sustained components after 10 mins of HgCl_2_ exposure in control neurons was 20.4 ± 2.8 mV (n = 7). The increased amplitude of membrane potential was significantly reduced to 6.86 ± 1.3 mV (n = 5) in the presence of MK 801. In summary, HgCl_2_ increased synaptic current inputs and caused enhancement of membrane discharges in pyramidal cortical neurons, and this increase in synaptic activity could be substantially attenuated by NMDA receptor block.

### Inhibition of NMDA receptor activity prevents mercury-induced impairment of network integrity and disruption of cytoskeleton component in cortical neurons

**Figure 6:**
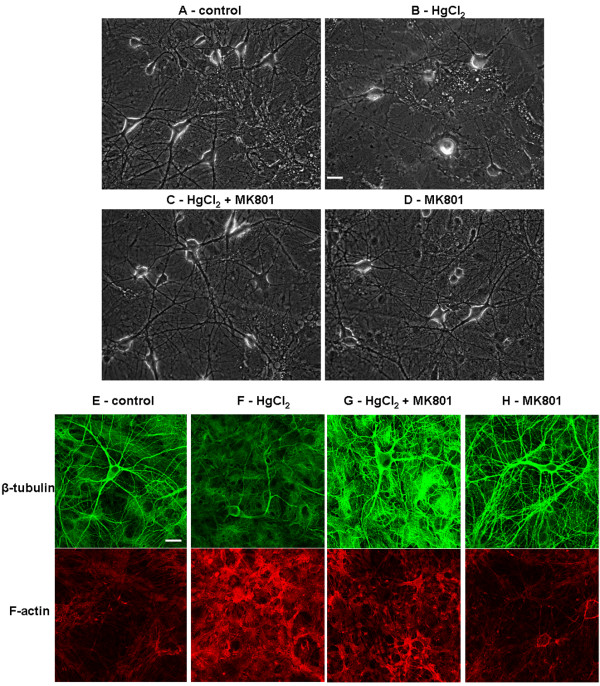
**Inhibition of NMDA receptor by MK 801 prevents HgCl**_**2**_**-induced disruption of neuronal network and degradation of cytoskeleton components.** To evaluate the protective effect of MK 801 on HgCl_2_-induced neurotoxicity, cells were grown for about 10 days and allowed to extend neurites and form network. Cultures were then maintained in the following conditions: control (**A**), 25 μM of HgCl_2_ (**B**), 25 μM of HgCl_2_ and 5–10 μM of MK 801 (**C**), and MK 801 alone (**D**). Phase contrast images were taken 16–24 hr later. Cells were subsequently fixed and stained with antibodies against β-tubulin and F-actin (**E**-**H**). Neurons exposed to HgCl_2_ exhibited retraction of neurite processes (**B**) and lower fluorescence intensity of β-tubulin (***F***) as compared with control cultures (**A** &**E**). HgCl_2_ in the presence of MK 801 failed to induce disturbance of network integrity and cytoskeleton staining (**C** &**G**). MK 801 alone did not affect neuronal network and fluorescence intensity of cytoskeleton staining (**D** &**H**). Scale bars, 25 μm.

Perturbation of synaptic activity or Ca^2+^ homeostasis, especially excessive loading of cells with Ca^2+^, activates a series of downstream cascades. These steps include proteinases, reactive oxygen species, and mitochondrial failure, which are lethal to cell cytoskeleton components or DNA structures [51-53]. Based on our results showing that MK 801 could significantly inhibit HgCl_2_-induced increases in synaptic activity and [Ca^2+^_i_, we predicted that MK 801 may exert a protective action on cortical neurons during exposure to HgCl_2._ To examine this, cells cultured for 10–12 days were exposed to the following four conditions: (1) control; (2) 25 μM of HgCl_2_; (3) co-application of HgCl_2_ at 25 μM and MK 801 at 5 or 10 μM, or following 30 mins of pretreatment with MK 801; (4) MK 801 alone for 16–24 hrs. Phase contrast pictures were taken the next day. We found that pretreatment or co application of MK 801, at both 5 and 10 μM, prevented HgCl_2_ - induced disruption of neuronal network. Specifically, Figure 6 shows that control neurons exhibit healthy cell bodies with well-established networks (A). HgCl_2_ alone severely disrupted neuronal network formation and triggered cell apoptosis (B). However, cells exposed to both MK 801 and HgCl_2_ still exhibited healthy morphologies with extensive neurite outgrowth and integrated network (C). MK 801 alone did not cause damage to neurons and their networks (D).

To further evaluate the protective action of MK 801 on HgCl_2_-induced neurotoxicity, we investigated whether MK 801 could also prevent HgCl_2_-induced damage to cytoskeleton components. To test this, cortical neurons were exposed to four conditions overnight as listed above, and cells were then fixed and stained with antibodies against β-tubulin and F-actin the next day. Confocal images showed that the fluorescence intensity of β-tubulin but not F-actin was substantially reduced in cells treated with HgCl_2_ alone (Figure 6F) as compared with control (Figure 6E). This result is consistent with what we observed previously (see Figure 3). Cells in dishes con-taining MK 801 at 5 μM and HgCl_2_ at 25 μM exhibited intense fluorescence for both β-tubulin and F-actin (Figure 6G). MK 801 alone did not affect staining in both instances (Figure 6H). In fact, the fluorescence intensity of F-actin in cells exposed to HgCl_2_ was even stronger in some cases as shown in Figure 5F & G.

In summary, our data indicate that an inhibition of NMDA-receptor activity indeed attenuates the degenerative effects of HgCl_2_ on cultured cortical neurons. These data further support the notion that NMDA synaptic transmission or receptor function plays an essential role in initiating HgCl_2_-induced toxicity in the CNS.

## Discussion

This study is the first to investigate HgCl_2_ neurotoxicity on rat cortical neurons from a variety of aspects – ranging from neuronal morphology to excitability. Specifically, our data demonstrate that HgCl_2_ elicits detrimental effects on young, developing cortical neurons – ranging from reduced cell survival, impaired neurite outgrowth, compromised network assembly to the degene-ration of mature networks. Importantly, our data caution against the possible detrimental effects of mercury at low nanomolar levels (sub-lethal doses). An apparent lack of short-term (3 days) or acute (< 24 hrs) effect of HgCl_2,_ at 25 and 100 nM, on cortical cell via-bility does not necessarily rule out cellular toxicity, as the detrimental changes may either be subtle or require longer exposures. In fact, HgCl_2_ at 100 nM has been demonstrated to cause remarkable disruption of neurite membrane integrity, and induce robust retraction and collapse of growth cones in *Lymnaea Stagnalis* neurons [39]. Low nanomolar concentrations of mercury have also been shown to affect the production of pathological hall-marks of Alzheimers’ diseases in cultured neurons [40,54] as well as affect cardiac function [55,56]. Therefore, a detailed evaluation of the effect of HgCl_2_ at these concentrations may still be required. Specially, the effect of HgCl_2_ on the motile structure of growth cones, the length of neurite elongation, and cell viability would need to be monitored using proper neuronal model types in which these parameters can be easily measured as shown in our previous study [57].

Mercury neurotoxicity has been implicated to involve myriad mechanisms and cellular targets. These include perturbation of Ca^2+^ homeostasis, dysfunction of mitochondria, glutamatergic excitability, disruption of cytoskeleton structures, reactive oxygen species (ROS), and many others [22,28,58]. However, the steps that initiate, mercury-induced neuronal degeneration and the underlying mechanisms remain largely unknown. In this study, we first explored the potential involvement of Ca^2+^ in mercury toxicity because Ca^2+^ is an important integrator of neuronal viability and excitability. The proper regulation of intracellular Ca^2+^ level plays an important role in regulation of growth cone motility, neurite initiation and outgrowth [59-61]. Intracellular Ca^2+^ levels either fall below or rise significantly above an optimal range have been shown to inhibit both of the growth cone motility and neurite outgrowth [59,62-64]. The data presented in this study show that HgCl_2_ triggered a sustained rise in [Ca^2+^_i_ in all cortical neurons examined and this effect on Ca^2+^ is not reversible (data not shown). The sustained elevation of [Ca^2+^_i_ in cortical neurons by HgCl_2_ may thus last for a long term and reach a level which would be detrimental for growth cone motility, neurite outgrowth and hence the network assembly during early neuronal development. Our data further demonstrated that HgCl_2_ also induced degradation of mature neurite and network connectivity (Figure 3 &6) suggesting that neuronal ultrastructure components such as cytoskeleton proteins may also be affected by HgCl_2_ –induced disturbance in Ca^2+^ homeostasis. In support of this hypothesis, our study demonstrates that HgCl_2_ indeed induced disassembly of cytoskeleton, primarily the β-tubulin proteins in cortical neurons. Our findings are consistent with previous studies showing that mercury induce disintegration of β -tubulin protein in several other species [39,65-67]. Neuronal cytoskeletal proteins have been shown to be extremely sensitive to intracellular Ca^2+^ levels and their assembly and disassembly can be influenced by Ca^2+^ either directly or indirectly via regulation of cytoskeleton associated proteins such as tau, a tubulin binding protein [68,69]. For instance, studies have shown that experimentally-induced, sustained elevation of [Ca^2+^_i_ either by Ca^2+^ ionophores, or depolarizing agents causes hyperphosphorylation of tau resulting in microtubule depolymerization and neuronal degeneration in cultured human cortical neurons [69]. As the microtubule cytoskeleton forms the basis for not only the structural integrity, but also for functional communications between neurons, the damage to the microtubules may result in abnormal physiological functions of the brain and hence aberrant animal behaviors.

Because Ca^2+^ is also an important regulator of cell excitability and gene expression, its entry (via NMDA receptors)-mediated synaptic activity has been shown to play crucial roles in neuronal development, synaptic plasticity, cell survival, and synaptic circuitry refinement [47,48,50]. Disturbance of neuronal activity even within one element of the network has been found to perturb the development of the entire circuitry and its physiological functions [47,50]. Considering the predominant effects of HgCl_2_ on NMDA receptor-mediated synaptic current inputs and membrane discharges in pyramidal cells, we postulated that Ca^2+^ entry via NMDA receptors may alter the membrane excitability and cellular signaling in pyramidal neurons resulting in a deficit in overall network activity and/or Ca^2+^ homeostasis. This is supported by our observations that HgCl_2_-induced rise in Ca^2+^ occurred almost simultaneously in a group of neurons within a network. The disturbance of Ca^2+^ homeostatic or other cellular signaling pathways resulting from an over stimulation of NMDA receptors might ultimately lead to, cytoskeleton disruption and cell death which are hallmarks of HgCl_2_-induced glutamatergic excitoxicity. Future studies are however required to decipher the precise involvement of NMDA receptors by HgCl_2_. In regard to the possibility of mercury directly modulating NMDA receptor function, the sulfhydral group in cysteine residues of NMDA receptors has been found to be the potential targets for regulation of NMDA receptor function by redox or alkylating agents. Oxidizing agents are known to decrease while sulfhydryl reducing agents potentiate NMDA receptor activity [70,71]. For instance, Tang and Aizenman (1993) demonstrated that alkylation of the NMDA redox site by the sulfhydryl alkylating agent N-ethylmaleimide (MEM) enhanced NMDA receptor response in cortical neurons [72]. Based on the fact that HgCl_2_ is a small molecular as MEM and has high affinity to sulfhydryl group, it is possible that HgCl_2_ may potentiate NMDA receptor function by directly interacting with its cysteine residue sulfhydryl groups, rendering the NMDA receptor dysfunctional. These postulates however need to be determined experimentally.

## Conclusions

Taken together, our data demonstrate that pre exposure of cells to MK 801 could remarkably inhibit HgCl_2_-induced increase in neuronal excitability, Ca^2+^ rise, and importantly prevent the damage to cytoskeleton components and network integrity. Our data suggest that over activation of NMDA receptors is likely the initial trigger for the downstream of cascades involved in HgCl_2_-induced toxicity. Inhibition of NMDA receptor activity is a potential and effective way to block the initiation of injurious effect of mercury in central neurons.

## Methods

### Cortical neuron cultures

All animal procedures were approved by the University of Calgary institutional animal use and care committee. Conditions were met with the standards established by the Canadian Council on Animal Care. The culture of cortical neuron was made using Sprague–Dawley rat pups on the day they were born. Dissociated cortical neurons were plated onto cover slips coated with poly-D-lysine (30 μg/ml, Sigma P6407) and laminin (2 μg/ml, Sigma L2020). Cortical neurons were cultured in neurobasal medium (Invitrogen, no. 21103–049) supplemented with 2% B27 (Invitrogen, no. 17504–044), L-Glutamine (200 mM) (Invitrogen, no. 25030–081), 4% FBS (Invitrogen, no. 12483–020), and penicillin-streptomycin (Invitrogen, no. 15140–122). Approximately 80% of the culture media was replaced every 3–4 days. Cultures were maintained at 37°C in an incubator circulated with air and 5% carbon dioxide. For counting studies, cortical neurons were plated onto cover slips with grids (Bellco Glass, no. 1916–91818).

To study the effect of Mercury (II) Chloride (HgCl_2_) on neuronal process initiation, neurite outgrowth and network formation, freshly dissociated cortical neurons were cultured either in the absence (control) or presence of different concentrations of HgCl_2_ (25 nM, 100 nM and 25 μM) for 3 days. Neurite outgrowth, cell viability and network formation were evaluated on day 3. To examine the acute effect (up to 24 hrs) of HgCl_2_ on newly established neuronal processes and network, cells were first cultured for 4 days to allow for the establishment of neurite outgrowth and network. Neurons were then exposed to HgCl_2_ at different concentrations overnight, and the effects of HgCl_2_ were examined the next day. To study the chronic effect (7 days exposure) of HgCl_2_ on well-established mature network, 10 day-old cells cultured on cover slips with grid were exposed to different HgCl_2_ concentrations and the effect was evaluated on day 17.

### Live/dead cell viability assay for cortical neurons

To determine and quantify the impact of HgCl_2_ on cell viability, cortical cultures that were maintained in control or drug-treated conditions were subsequently loaded with the LIVEDEAD® ViabilityCytotoxicity Kit (Molecular probes, L-3224) for 30 mins at room temperature (21-22°C). This two-color assay was developed based on the fact that intracellular esterase activity and an intact plasma membrane are unique characteristics of live cells. The LIVEDEAD® ViabilityCytotoxicity Kit discriminates live from dead cells by simultaneously staining with green-fluorescent calcein-AM to indicate intracellular esterase activity and red-fluorescent ethidium homodimer-1 to indicate loss of plasma membrane integrity. Preparations were visualized using confocal microscopy (LSM 510 Meta, Zeiss, Germany) under a 10x or 20x objective at 488 nm excitation (green) and 548 nm (red) wavelength and images were collected using a band pass filter (560–615 nm). The number of cells labeled with both colors was subsequently counted using imageJ software.

### Whole-cell patch-clamp recordings in cortical neurons

Whole-cell recordings of synaptic inward currents and membrane potentials were performed on pyramidal cortical neurons after 7 to 15 days in culture at room temperature (21-22°C) using a Multiclamp 700B amplifier (Axon Instruments; Sunnyvale, CA, USA) connected to an analog-to-digital interface Digidata 1322 (Axon Instruments). Signals were acquired and stored through pClamp 9.2 software (Axon Instruments). Synaptic currents were recorded under voltage-clamp mode with the holding potential of −70 mV throughout. Membrane potentials of pyramidal cells were recorded under current-clamp mode. The external solution contained (in mM) NaCl, 135; CaCl_2_, 3; KCl, 5; MgCl_2_, 2; HEPES, 10; D-Glucose, 10; pH adjusted to 7.3 with NaOH. The internal pipette solution was composed of (in mM) K^+^-Gluconate, 130; NaCl, 7; MgCl_2_, 0.3; HEPES, 10; EGTA, 0.1, ATP-Mg, 3; GTP-Na, 0.6; pH adjusted to 7.3 with KOH. The osmolarity for internal solution was approximately 300 mOsm (295–305) and for external solution approximately 330 mOsm (320–340). Borosilicate patch pipettes (A-M Systems, Inc; Sequim, WA, USA) were pulled using a horizontal micropipette puller (Model P-97, Sutter instrument Co., USA) and had a tip resistance ranging from 3–5 MΩ after filling with internal solutions. Only cells with series resistances less than ~15 MΩ and leaks less than ~100 pA were selected for the analysis. Data were analyzed using Clampfit 9.0 software (Axon Instruments), and traces were plotted using OriginPro 8.0 SRO (OriginLab Corporation; Northampton, MA, USA).

### Ca^2+^ Imaging

Fura-2 acetoxymethyl ester (AM) ratiometric Ca^2+^ imaging experiments were performed to study the action of HgCl_2_ and/or N-methyl-D-Aspatate (NMDA) receptor activity on intracellular Ca^2+^ level in cortical neurons. Specifically, neurons that were cultured for 5 to 10 days were loaded with the membrane permeable and ratiometric Ca^2+^ sensor Fura-2 AM (Molecular Probes, Carlsbad, CA) at 5 μM for 30 mins at 37°C. Cells were subsequently washed three times with normal saline solution containing 0 Mg^2+^ and 3 μM glycine to activate NMDA receptor at resting level. The composition of normal saline included (in mM): NaCl, 135; CaCl_2_, 3; KCl, 5; HEPES, 10; D-Glucose, 10; pH adjusted to 7.3 with NaOH. Culture dishes were then mounted on an inverted microscope and neurons were exposed to excitation wavelengths of 340 and 380 nm delivered using a LAMBDA DG4 high-speed wavelength switcher (Sutter Instrument, Novato, CA) through a 40x oil objective. The emitted fluorescence signal was collected at 510 nm by a Regina Exi camera. Images were acquired with the Northern Eclipse software ionwave program (Empix Imaging, Canada). The change in intracellular Ca^2+^ level was reflected by the ratio of fluorescence intensity obtained at 340 nm and 380 nm.

### Immunochemistry and confocal microscopy

Cultured cells were fixed for 1 h with pre-warmed 4% paraformaldehyde and subsequently washed four times with 1x PBS. Cell were permeabilized for 1 h with incubation media (IM) (0.5% Triton in 1x PBS with 10% goat serum) and then incubated overnight with monoclonal anti-β-tubulin antibody produced in mouse (1:500) (Sigma, T0198). Cells were rinsed two times with 1x PBS the next day. Cells were then incubated with AlexaFluor 488 goat anti-mouse Ig6 secondary antibody (1:100) (Invitrogen, A-11001) for 1 h at room temperature (21-22°C) under dark conditions. Cultures were subsequently rinsed two times with 1x PBS and incubated for 30 minutes with rhodamine phalloidin (1:20) (Invitrogen, no. R415). Following two time wash with 1 x PBS and one quick rinse with double distilled H_2_O cover slips were mounted using MOWIOL mounting media with 4’6-diamidino-2-phenylindole dihydrochloride (Sigma-Aldrich). Samples were viewed using confocal microscopy (LSM 510 Meta, Zeiss, Germany) under a 20x dry or 40x water objective at 488 nm excitation (green, β-tubulin) and 548 nm (red, F-actin) wavelength. Images were collected using a band pass filter (560–615 nm). To assess the level of ß-tubulin and F-actin staining, image acquisition parameters for control and drug-treated neurons were kept the same.

### Chemicals

All chemicals including Mercury (II) chloride (HgCl_2_, M1136) and MK 801 (M107) were purchased from Sigma-Aldrich (Oakville, Ontario, Canada). Fura-2 acetoxymethyl ester (AM) was purchased from (Molecular Probes, Carlsbad, CA). HgCl_2_ was dissolved in deionized water (for electrophysiological and Ca^2+^ imaging experiments), or in culture medium (for toxic study) to a stock concentration of 10 mM or 10 μM. The applied solutions (0.025-25 μM) were diluted with normal external solution or culture medium.

### Statistical analysis

Data were analyzed statistically using Student’s *t* test or one-way analysis of variance (ANOVA) as appropriate. Post hoc analysis was conducted using Tukey’s test. Values were considered statistically significant at the level of P < 0.05. The data are presented as mean ± S. E.M. Each experiment was replicated a minimum of four times; the actual number of replicates for each experiment is listed in the corresponding figure legend or in the text.

## Competing interests

The authors declare that they have no competing interests.

## Authors’ contributions

FX, SF, SS, FXZ and LC conducted experiments and data analyses. Specifically, FX, SF, SS, LC performed the toxicity and cell viability study. FX and FXZ conducted the calcium imaging and electrophysiological experiments. FX, NIS and GWZ drafted and edited the manuscript. All authors read and approved the final manuscript.
